# Childhood maltreatment is associated with cortical thinning in people with eating disorders

**DOI:** 10.1007/s00406-022-01456-y

**Published:** 2022-07-19

**Authors:** Giammarco Cascino, Antonietta Canna, Andrea Gerardo Russo, Francesco Monaco, Fabrizio Esposito, Francesco Di Salle, Palmiero Monteleone, Alessio Maria Monteleone

**Affiliations:** 1 Department of Medicine, Surgery and Dentistry ‘Scuola Medica Salernitana’, Section of Neurosciences, University of Salerno, Via Allende 1, Baronissi, 84081 Salerno, Italy; 2 Department of Advanced Medical and Surgical Sciences, University of Campania “Luigi Vanvitelli”, Naples, Italy; 3 Department of Mental Health, ASL Salerno, Salerno, Italy; 4 Department of Psychiatry, University of Campania “Luigi Vanvitelli”, Naples, Italy

**Keywords:** Eating disorders, Cortical thickness, Childhood maltreatment, Neurodevelopment

## Abstract

Childhood maltreatment (CM) is a non-specific risk factor for eating disorders (ED) and is associated with a greater severity in their clinical presentation and poorer treatment outcome. These data suggest that maltreated people with ED may be biologically other than clinically different from non-maltreated people. The aim of the present study was to investigate cortical thickness (CT), a possible biomarker of neurodevelopment, in people with ED with or without history of CM and in healthy women. Twenty-four healthy women, 26 with anorexia nervosa and 24 with bulimia nervosa underwent a 3T MRI scan. All participants filled in the childhood trauma questionnaire. All neuroimaging data were processed by FreeSurfer. Twenty-four participants with ED were identified as maltreated and 26 participants with ED as non-maltreated. All healthy women were non-maltreated. Compared to healthy women, maltreated people with ED showed lower CT in the left rostral anterior cingulate gyrus, while compared to people with ED without history of CM showed lower CT values in the left superior frontal and in right caudal middle frontal and superior parietal gyri. No significant differences emerged in CT measures between healthy women and people with ED without history of CM. The present findings show for the first time that in adult people with ED childhood maltreatment is associated with cortical thinning in areas implicated in the modulation of brain processes that are acknowledged to play a role in the psychopathology of ED.

**Supplementary Information:**

The online version contains supplementary material available at 10.1007/s00406-022-01456-y.

## Introduction

Early traumatic experiences are recognized as non-specific risk factor for the onset of various psychiatric disorders [1–3]. These experiences include both childhood abuse and neglect [4] and produce physiological and neurohumoral reactions that may affect brain development with possible psychopathological consequences in genetically vulnerable individuals [5–7]. Other than increasing the risk of onset of various psychiatric disorders, childhood maltreatment (CM) may allow to clinically and biologically distinguish individuals with the same psychiatric disorder. Indeed, for example, maltreated individuals with different psychiatric disorders have been proved to show earlier age at onset, greater symptom severity, more comorbidity, greater risk for suicide, and poorer treatment outcome than non-maltreated people [1]. Moreover, maltreated individuals with schizophrenia, other psychotic disorders, major depression or antisocial personality disorder presented neuroanatomical differences with respect to healthy controls in terms of reduced volume of anterior cingulate cortex (ACC) and dorsolateral prefrontal cortex (PFC), whereas non-maltreated individuals with the same psychiatric diagnosis did not [8–10]. All these findings support the existence of a “maltreated ecophenotype” biologically and clinically different from the non-maltreated one [1, 2].

People with eating disorders (ED) exhibit a prevalence of CM higher than general population [11] and, as for other psychiatric conditions, a history of CM in the context of ED has been found associated with an earlier age at onset, a greater clinical severity, a more frequent comorbidity with other psychiatric conditions [12] and a poorer treatment response [13]. Neuroendocrine modifications [14] as well as a heightened biological and emotional vulnerability to acute social stressor exposure [15] have been reported in people with ED and history of CM. This evidence suggests the possibility to identify a “maltreated ecophenotype” also in people affected by ED.

To our knowledge, only one study investigated the association between CM and brain morphology in people with EDs and found a reduced gray matter volume in the right paracentral lobule and in the left inferior temporal gyrus of maltreated patients [16]. However, cortical thickness (CT), which reflects the number of neurons within cortical columns [17] and is believed to represent a measure of the neurodevelopment process more specific and biologically more significant than brain volume [18], although explored in people with ED, has been never assessed in relation to the individual history of CM. Indeed, several studies lead to the conclusion that cortical thinning is frequently observed in the acute phase of ED and is related to malnutrition, especially in anorexia nervosa (AN) [19–21]. However, other studies have reported the persistence of cortical thinning in weight-recovered people with AN [22] or a higher CT in underweight people with AN compared to healthy controls [23]. In people with bulimia nervosa (BN), there were findings of both higher and lower CT values compared to healthy control in frontal and temporo-parietal areas [20, 21]. Other than the heterogeneity of the studies samples, a further variable that can influence CT in people with ED and explain part of the literature inconsistency could be a history of CM. Therefore, the aim of the present study was to identify and estimate specific variations in CT associated with a history of CM in people with ED. Based on previous findings in maltreated individuals [8–10], we hypothesized that people with ED and history of CM should show a cortical thinning with respect to both people with ED without history of CM and to a group of age- and sex-matched healthy controls.

## Materials and methods

### Subjects and clinical assessment

Study participation was proposed to patients consecutively admitted to the adult ED outpatient centers of the Departments of Psychiatry of the University of Campania “Luigi Vanvitelli” and the University of Salerno. Participants with ED had to meet the following inclusion/exclusion criteria: (1) diagnosis of current AN or bulimia nervosa (BN), according to DSM-5, confirmed by the Structured Clinical Interview for DSM-5 Disorders—Research Version [24]; (2) age ≥ 18 years; (3) absence of severe physical disorders or current comorbid psychiatric disorders; (4) no history of psychoactive substance use or head trauma; (5) no use of medications or oral contraceptives in the past 2 months; (6) right handed; (7) willingness to cooperate in the experimental procedures and to sign a written informed consent. Diagnostic assessment and collection of sociodemographic and clinical data were performed by trained psychiatrists.

Healthy women were recruited through advertisements on the campus of the University of Salerno. They had to be drug-free and physically and mentally healthy, as assessed by a routine physical examination and the Mini International Neuropsychiatric Interview [25]. All subjects gave their written consent after being fully informed of the nature and procedures of the study. The study was approved by the Ethics Committee of the University of Campania “Luigi Vanvitelli” and performed in accordance with 1964 Declaration of Helsinki and its later amendments.

All the participants completed the short form of the Childhood Trauma Questionnaire (CTQ) [26] to evaluate childhood trauma exposure. The CTQ is a 28-item questionnaire investigating childhood experience across five types of CM: emotional neglect, emotional abuse, sexual abuse, physical neglect and physical abuse. For each subscale, validated cutoff scores indicating the occurrence of maltreatment have been provided [27]: emotional neglect ≥ 15, emotional abuse ≥ 10, sexual abuse ≥ 8, physical abuse ≥ 8, physical neglect ≥ 8. We classified as “maltreated” (Mal) participants who scored higher than the threshold in at least one subscale and as “non-maltreatment” (noMal) those who scored below the threshold for all five subscales.

### Image acquisitions

All subjects underwent a 3T scanner (MAGNETOM Skyra, Siemens, Erlangen, Germany). The image protocol consisted of the acquisition of T1-weighted 3D Magnetization Prepared RApid Gradient Echo (MPRAGE), sagittal orientation, matrix size 256 × 240, FOV 240 × 256 mm, 136 slices, slice thickness 1.2 mm, in-plane voxel size 1 × 1 mm, flip angle 9°, TR/TE 2300/2.98 ms, one average.

All participants with ED were outpatients and underwent the MRI scanning within two weeks from the first assessment, before starting any type of treatment. Participants from the control group and menstruating patients were tested in the follicular phase of their menstrual cycle.

### Processing and measurements of cortical thickness

All data were processed using FreeSurfer (FS) version 6.0 (https://surfer.nmr.mgh.harvard.edu/), on Hewlett-Packard workstation equipped with an Intel^®^ CoreTM i5-4590S CPU @3.00 GHz and 8 GB of RAM and running Linux Ubuntu 20.04 LTS.

Raw T1w images of all subjects were imported in FS and submitted to the standard structural image preprocessing and reconstruction pipeline of FS via the “recon-all" command (for a detailed description of this procedure please see https://surfer.nmr.mgh.harvard.edu/fswiki/ReconAllTableStableV5.3) [28–35].

Preprocessed data were visually inspected and corrected to remove non-gray matter tissues (e.g. dura mater and blood vessels) incorrectly included into the gray matter volume.

After this editing step the “autorecon-pial” command of the FS pipeline was launched.

Each subject folder was used to extract the CT measurements. Maps of CT were computed in order to perform a vertex-by-vertex analysis.

### Statistical analysis

CT maps underwent a general linear model (GLM) analysis to evaluate differences in the following contrasts: HC vs maltreated people with ED, maltreated people with ED vs non-maltreatment people with ED and HC vs non-maltreatment people with ED. Age and body mass index (BMI) were included as nuisance covariates. Statistical maps were then corrected for multiple comparisons using Monte Carlo simulation with 10,000 iterations and a statistical threshold of *p* = 0.05. We extracted from each cluster showing significant differences the mean CT values to plot the trend of these measures across groups. Then, because it has been shown that CT may have a nonlinear relationship with age [36], a generalized linear model was performed including linear and quadratic age effects.

Differences in demographic and clinical variables among the groups were tested by means of one-way analysis of variance (ANOVA) followed by the post hoc Tukey’s test.

## Results

Twenty-four healthy women, 26 with AN (19 restrictive subtype and 7 binge-purging subtype) and 24 with BN participated in the study. Based on CTQ cutoff scores, 12 participants with AN and 12 with BN were identified as maltreated and 14 participants with AN and 12 with BN as non-maltreatment. All healthy women were “non-maltreated.” Therefore, participants were split in 3 groups: 26 maltreated participants with ED, 24 non-maltreatment participants with ED and healthy control (HC). The groups did not differ in age, age at onset and illness duration, while healthy women showed a BMI significantly higher than participants with ED both with and without history of childhood maltreatment (Table 1). Twenty-four patients with a diagnosis of AN (12 maltreated and 12 not-maltreatment) were amenorrheic. The mean duration of amenorrhea was 5.9 ± 4.2 years. Fifteen participants with ED (30%) disclosed emotional neglect, 16 (32%) emotional abuse, 5 (10%) sexual abuse, 8 (16%) physical neglect, 7 (14%) physical abuse. Among maltreated people with ED, 10 (38.5%) reported only one type of CM, while 16 participants (61.5%) reported 2 or more different types of CM.

**Table 1:** Demographic characteristics of eating disorder (ED) women with (Mal) or without (noMal) history of childhood maltreatment and healthy control (HC) women

	ED Mal (*n* = 26)	ED No Mal (*n* = 24)	HC (*n* = 24)	*F*	*df*	*p*
Age, years	30.12 ± 10.25 (18|48)	27.88 ± 8.45 (18|48)	26.21 ± 4.49 (18|42)	1.45	2,71	0.24
BMI, kg/m^2^	18.85 ± 3.07 (14.0|28.7)	18.19 ± 5.28 (13.7|24.8)	21.88 ± 2.33 (18.6|28.1)	6.62	2,71	0.002*
Age at onset, years	17.80 ± 4.05 (13|30)	18.95 ± 4.98 (12|30)		0.75	1,47	0.39
Illness duration, years	13.20 ± 8.39 (1|35)	9.41 ± 9.11 (2|29)		2.21	1,47	0.14

Measures are expressed as means and standard deviations (min|max)

*BMI* body mass index; *ED* eating disorder; *mal* maltreated; *HC* healthy controls

*Tukey’s post hoc: HC > ED Mal, ED No Mal

**Fig. 1:**
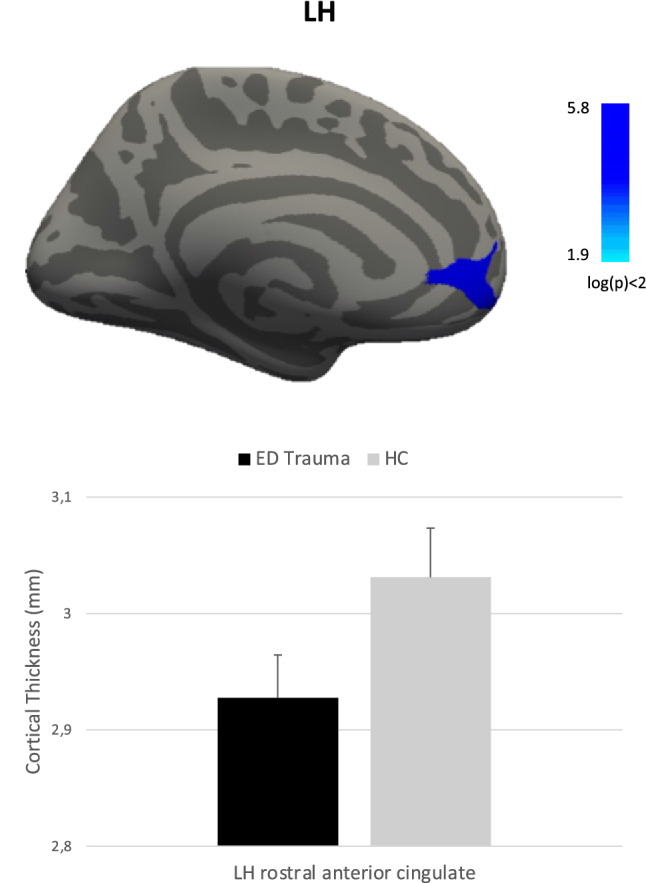
Differences in cortical thickness between maltreated patients with eating disorders and healthy controls. *LH* left hemisphere

**Fig. 2:**
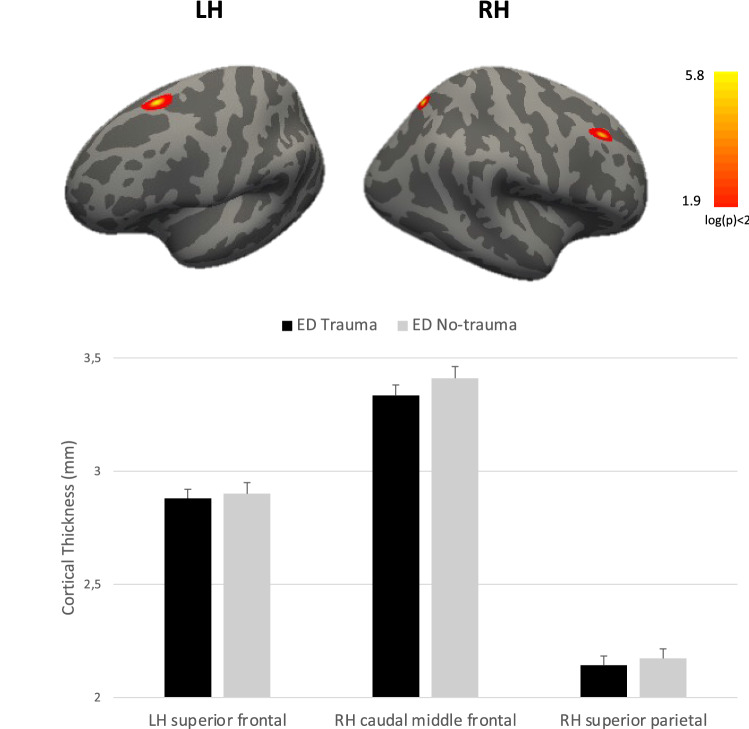
Differences in cortical thickness between maltreated and non-maltreated patients with eating disorders. *LH* left hemisphere; *RH* right hemisphere

Compared to HC, maltreated people with ED showed lower CT values in the left rostral anterior cingulate gyrus (Fig. 1), while compared to non-maltreatment people with ED showed lower CT values in the left superior frontal, in right caudal middle frontal and in right superior parietal gyri (Fig. 2). No significant differences emerged in CT measures between HC and non-maltreatment people with ED. Table 2 shows the coordinates (in the Talairach space) of the peaks and the cluster sizes of significant clusters.

**Table 2:** Coordinates of the peaks and size of clusters that significantly differ in cortical thickness among the groups

Maltreated ED vs HC				
Area	Talairach coordinates (x, y, z)			Size (mm^2^)
Left rostral anterior cingulate	− 9.2	41.7	− 0.6	468.29

Maltreated ED vs No-maltreated ED				
Area	Talairach coordinates (x, y, z)			Size (mm^2^)
Left superior frontal	− 20.8	20.0	49.8	238.47
Right caudal middle frontal	33.2	23.2	42.5	254.59
Right superior parietal	17.1	− 64.2	51.6	232.15

Results from generalized linear model including both linear and quadratic effects of age are reported in supplementary materials.

## Discussion

According to our study hypothesis, maltreated people with ED showed a significant cortical thinning compared to non-maltreatment people with ED and HC, whereas measures of CT in non-maltreatment people with ED did not differ significantly from those of HC. These results are in line with a large body of the literature, showing that CM is associated with cortical thinning in both clinical and non-clinical populations [37–40]. In our previous study we found a reduced cortical volume in the right paracentral lobule and in the left inferior temporal gyrus in maltreated people with ED compared to non-maltreatment ones [16], while in the present study, we have shown that CT is not affected by CM in those areas. Since CT contributes with cortical surface area in the determination of the cortical gray matter volume [41], the present findings support the idea that CM may be associated with a reduced cortical surface rather than cortical thinning in inferior temporal gyrus and paracentral lobule. However, this hypothesis needs to be confirmed.

Interestingly, the localization of cortical thinning in ACC and PFC is consistent with reports of previous studies in non-clinical populations with history of CM [37–39]. The ACC is the cortical region most frequently affected in maltreated individuals with findings of reduced volume [42], connectivity [43] and thickness [38]. This area plays a role in emotional regulation processes [44], self-awareness [45] and reward [46]. Emotion dysregulation [47] has been identified as a variable making emotionally or sexually abused individuals more vulnerable to EDs. Moreover, reflection on one’s own physical characteristics is a key feature of ED psychopathology [48], and it has been reported to mediate early life adverse experience and ED core psychopathology [49]. Finally, hypoactivation of brain areas of reward system has been found in people with an history of CM and in people with ED, even after recovery [50, 51]. These findings, taken together, not only support the involvement of reward mechanisms in the pathophysiology of ED, but they also suggest that dysregulation in reward mechanisms may be, at least in part, linked to CM [52, 53].

The detection of a significant reduction of ACC CT in maltreated people with ED compared to HC, but not with respect to non-maltreatment people with ED, suggests that structural alterations in ACC associated with history of CM may contribute to the greater severity of ED psychopathology of maltreated patients. Indeed, structural and functional differences in the cingulate cortex have been found related to disease severity in AN [54].

The finding of a reduced CT in frontal gyri in maltreated people with ED is consistent with a previous report of cortical thinning in lateral PFC in non-clinical adolescents with a history of CM [55]. The PFC is implicated in specific types of emotion regulation, particularly in response to social exclusion [56], and CM severity was associated with PFC responsivity to social exclusion in young adults [57]. Alterations in social functioning have been found in people with ED either before or after illness onset [58], and people with EDs showed blunted cortisol and heightened emotional responses to a psychosocial stressor [59]. People with an EDs showed vigilance to rejection and avoidance of social reward [60]. Moreover, attentional bias to rejecting faces was correlated with adverse childhood experiences [60].

Several limitations of this study need to be acknowledged. First, the relatively small size of our sample, although consistent with most of the neuroimaging studies in people with EDs [61], did not allow to identify differences between ED diagnostic groups and could be a possible confounding factor for the study findings. To overcome this issue, BMI and age, which can affect CT [62], were included as nuisance covariates in the GLM analysis.

Second, we did not take into account the possible association between different types of trauma and CT alterations. Thus, alterations reported in maltreated people with EDs likely reflect additive effects of several forms of maltreatment, which often co-occur [63]. Third, the lack of a non-clinical population with a history of childhood maltreatment did not allow us to disentangle the effect of psychiatric disorder from that of childhood maltreatment. However, the study design is consistent with those already conducted in clinical population with CM [9]. Finally, another limitation is the retrospective self-reports of childhood experiences which does not exclude recall bias. However, it has been showed that false negative reports are more frequent than false positive, leading to downward biases in estimated associations between CM and outcome variables [64].

In conclusion, the present findings show that in people with ED childhood maltreatment is associated with cortical thinning in several areas that could play a role in the onset and maintenance of EDs. Moreover, this study corroborated the hypothesis of the existence of a “maltreated ecophenotype,” which recommends grouping individuals affected by the same psychiatric condition into subgroups characterized by different clinical and biological correlates [65]. Further studies are needed to ascertain whether changes in CT in adult people with ED represent a biological marker of CM exposure and whether these alterations could mediate the risk of CM to develop an ED in the adulthood.

## Supplementary Information

Below is the link to the electronic supplementary material.

Supplementary file1 (DOCX 552 kb)
