# Supplementary material for: Childhood maltreatment is associated with cortical thinning in people with eating disorders
Source: Eur Arch Psychiatry Clin Neurosci. 2022 Jul 19;273(2):459–66. doi: 10.1007/s00406-022-01456-y (PMC10070200; doi:10.1007/s00406-022-01456-y)
Supplement: Supplementary file 1 — Supplementary file1 (DOCX 552 kb) [file 406_2022_1456_MOESM1_ESM.docx]

**Supplementary Materials**

In the contrast between maltreated people with ED and non-maltreatment people with ED the generalized linear model including linear and quadratic effect of age and body mass index as nuisance covariate showed significant lower values of cortical thickness in the left superior-frontal gyrus and right caudal middle frontal, supramarginal and superior parietal gyri of maltreated people with ED. In the contrast between healthy controls and maltreated people with ED, significant lower values of CT emerged in left rostral anterior cingulate and lateral occipital gyri and right inferior parietal gyrus of maltreated people with ED. No significant differences emerged in the contrast between non-maltreatment people with ED and healthy controls. Coordinates in the Talairach space of the peaks and the cluster sizes of significant clusters are reported in Supplementary Table 1. Significant clusters are shown in Supplementary Figures 1 and 2.

**Supplementary figure 1.** Differences in cortical thickness between maltreated and non-maltreatment patients with eating disorders. LH, left hemisphere; RH, right hemisphere.

**RH LH**


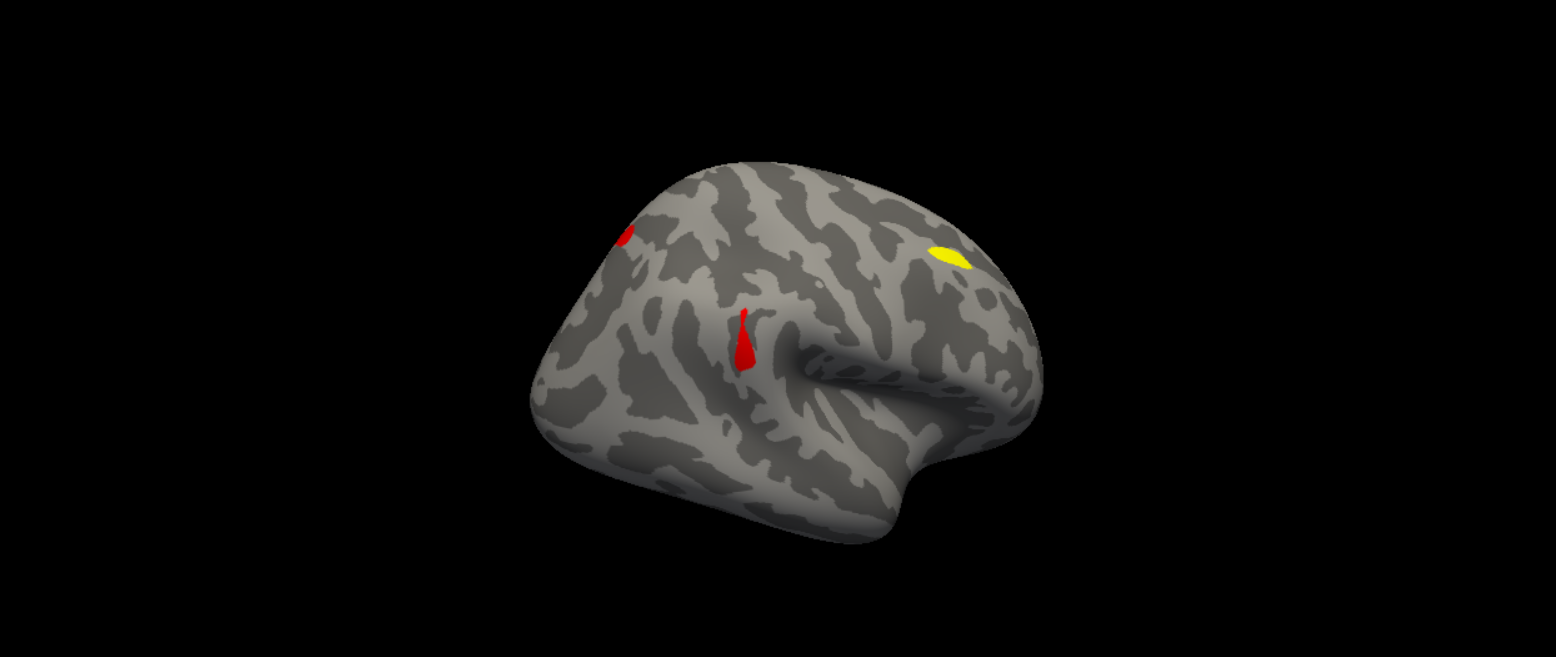

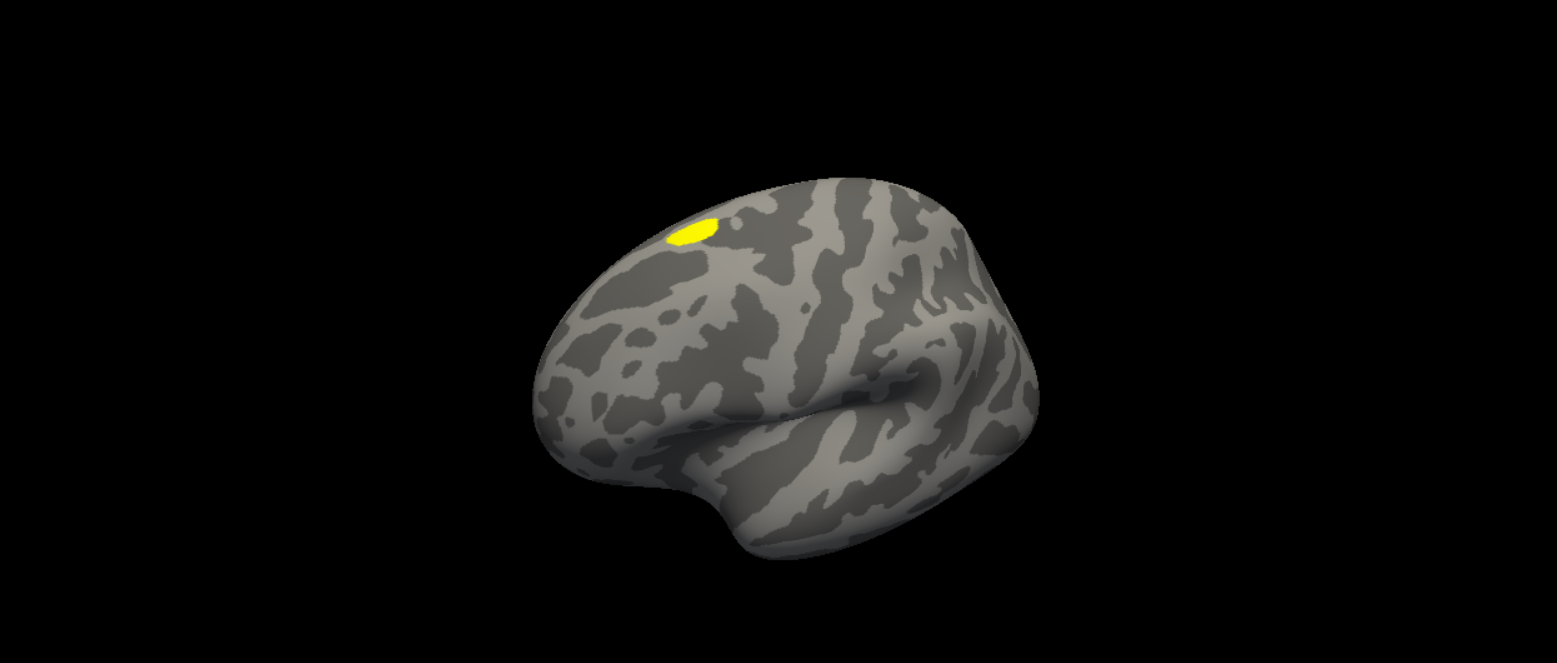


**Supplementary figure 2.** Differences in cortical thickness between maltreated patients with eating disorders and healthy controls. LH, left hemisphere; RH, right hemisphere.

**RH LH**


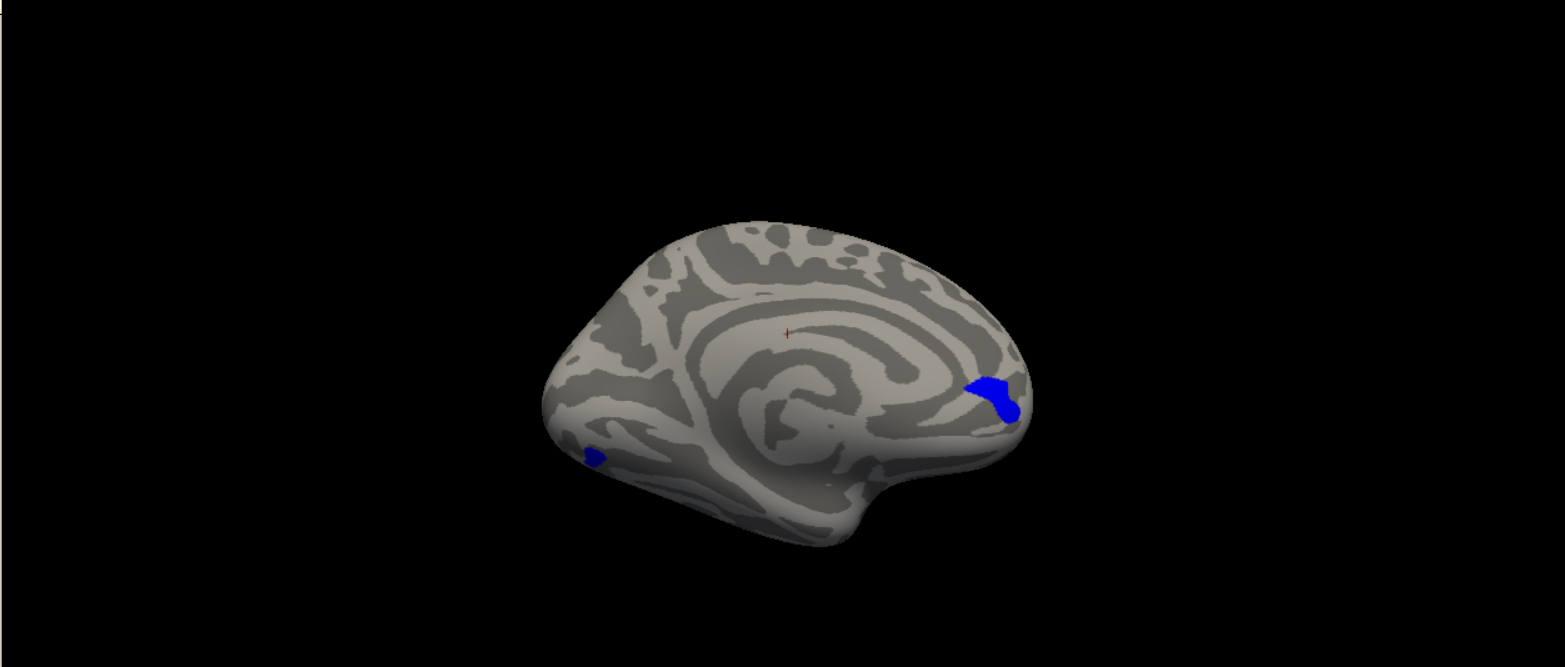

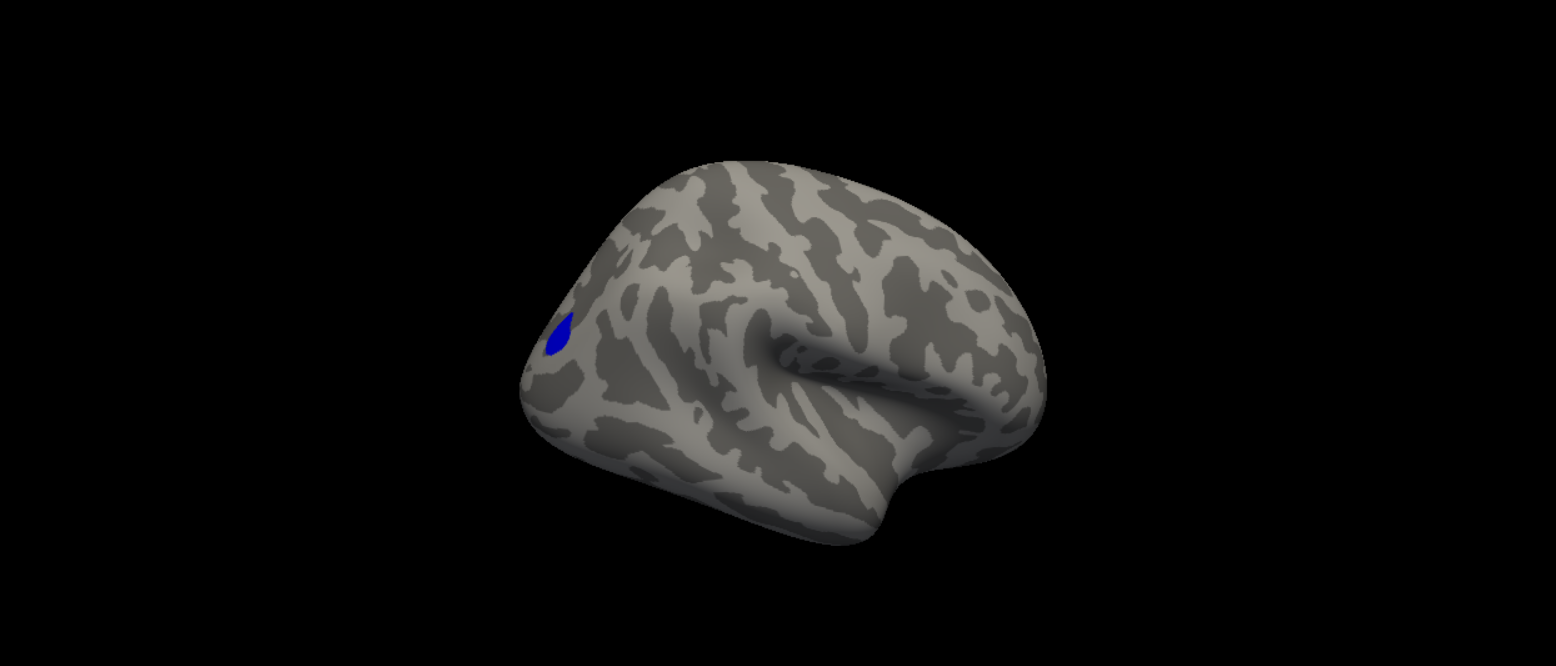


| **Supplementary Table 1.**  Coordinates of the peaks and size of clusters that significantly differed in cortical thickness among the groups. | | | | |
| --- | --- | --- | --- | --- |
| **Maltreated people with ED vs healthy Controls** | | | | |
| Area | Talairach coordinates (x,y,z) | | | Size (mm^2^) |
| left rostral anterior cingulate | -9.3 | 41.6 | -1.1 | 257.24 |
| left lateral occipital | -20.7 | -80.4 | -2.4 | 166.91 |
| right inferior parietal | 31.1 | -75.6 | 17.4 | 208.41 |
| **Maltreated people with ED vs No-maltreatment people with ED** | | | | |
| Area | Talairach coordinates (x,y,z) | | | Size (mm^2^) |
| left superior frontal | -20.8 | 19.5 | 49.4 | 215.17 |
| right caudal middle frontal | 33.7 | 22.9 | 42.7 | 240.34 |
| right superior parietal | 16.3 | -64.6 | 51.9 | 173.17 |
| right supramarginal | 55.6 | -41.3 | 24.6 | 157.93 |
